# Novel enrichment reduces boredom-associated behaviors in housed dairy cows

**DOI:** 10.3168/jdsc.2023-0475

**Published:** 2024-02-01

**Authors:** Alison L. Russell, Laura V. Randall, Nikki Eyre, Jasmeet Kaler, Martin J. Green

**Affiliations:** School of Veterinary Medicine and Science, The University of Nottingham, Sutton Bonington, Loughborough, LE12 5RD, United Kingdom

## Abstract

There is currently societal interest and concern for dairy cow welfare. It has been suggested that boredom poses a welfare issue for dairy cows, particularly when presented with extended disposable time in housed environments that lack stimuli. Farm animals experience a multitude of affective states, which has created a need for positive experiences to be included in welfare management. Environmental enrichment can reduce boredom and facilitate positive experiences; however, the research in cows is limited. To assess the behavioral impact of a simple enrichment on commercially housed dairy cows, we provided 24-h access to a novel object, for 3 wk, for 2 separate groups of cows. Two boredom-associated behaviors significantly decreased when the object was present compared with when it was not present: “idling” behavior and unsuccessful robotic milking attempts (“refusals”). In addition, there was a significant increase in the occurrence of self-grooming during treatment weeks, when the novel object was present. These results suggest that idling and refusals may be behavioral indicators of boredom in dairy cows.

## Highlights

• Cows had fewer robotic milking refusals during the period a novel object was present.
• Fewer idling events occurred during the period a novel object was present.
• Self-grooming appeared to increase with the use of novel enrichment.

## Graphical Abstract

**Figure:**
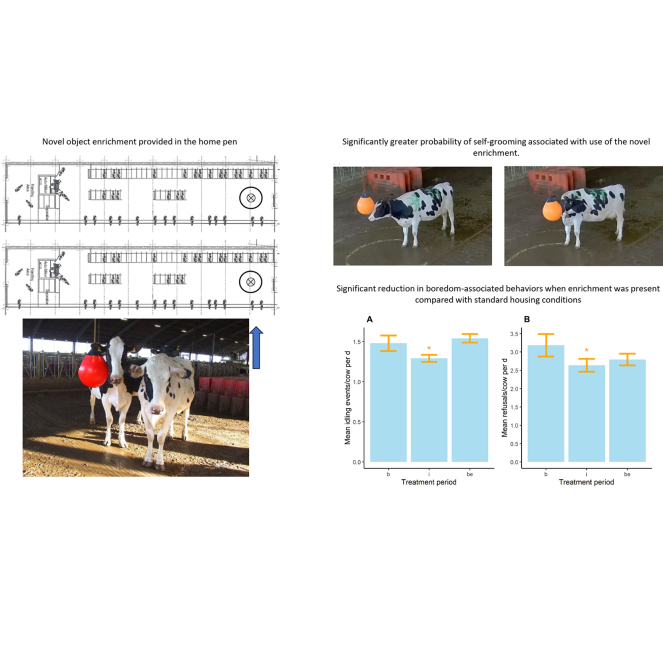
**Summary** Boredom has been suggested as a potential cause of suboptimal welfare for housed dairy cows; however, research evaluating boredom and the impact of environmental enrichment is lacking. This study investigated the effect that a novel object in the home environment had on the behavior of 2 groups of dairy cows (n = 71). Cows had fewer robotic milking refusals, and the occurrence of idling behavior was reduced when the novel object was present compared with when cows were housed in standard conditions. In addition, the occurrence of self-grooming increased while the novel object was present. After 3 weeks, 83% of cows were still using the novel object at least once each day. These results suggest that unsuccessful milking attempts and idling may be behavioral indicators of boredom in dairy cows and that additional sources of environmental enrichment may enhance the welfare of housed dairy cows.

Managing dairy cows indoors has increased in Great Britain, with only 1% of farms not housing their cows at any point throughout the year (March et al., 2014). Livestock housing is often considered barren, with limited space, limited opportunities for species-specific behavior, and monotonous conditions that predispose animals to experience boredom (Wemelsfelder, 1993; Mason and Burn, 2018). Boredom is a negative affective state in animals (Burn, 2017; Meagher, 2019), caused by an environment that provides fewer behavioral opportunities and experiences than an animal is motivated to have (Mason and Burn, 2011), and has been highlighted as a potential cause of suboptimal welfare for housed dairy cows (DeVries et al., 2007; Crump et al., 2019). There is no consensus on the definition of welfare, which is a complex topic in itself; however, at its simplest, it is generally now considered to be an animal's overall state of physical and psychological well-being.

Environmental enrichment, which is diversification of captive environments to improve well-being (de Azevedo et al., 2007), is usually the first approach for alleviating boredom in animals (Meagher, 2019), by providing environmental opportunities for control and exploration (Fraser et al., 1991). Research in different species has demonstrated reduced behavioral indicators of boredom in conditions which provide additional behavioral opportunities via enrichment (Wood-Gush and Beilharz, 1983; Meagher and Mason, 2012); however, scientific studies evaluating behavioral indicators of boredom in housed dairy cows are lacking.

Wakeful inactivity has emerged as a potential behavioral expression of boredom and other negative affective states in animals (Fureix et al., 2012; Fureix and Meagher, 2015). Animals housed in monotonous environments generally spend more time inactive than animals in more diverse, stimulus-varied conditions (Webb et al., 2017; Burn et al., 2020). Housed buffalos have been shown to display more idling behavior, one such form of wakeful inactivity, compared with buffalos provided with additional space and enrichment (Tripaldi et al., 2004; De Rosa et al., 2009). Increased idling behavior has also been observed in housed dairy cows without access to daily grazing (Di Grigoli et al., 2019) and in cattle housed on slats compared with cattle with exercise areas and outdoor space (Hintze et al., 2020).

Robotically milked cows choose when to be milked and are conditioned to voluntarily enter the robot, with concentrate fed during milking. Based on specific selection criteria such as a minimum milking interval or individual cow milk yield, a cow may be immediately released by the robot (without the provision of food); these are classed as refusals. This type of visit has been shown to make up 30% to 58% of the total visits to the robot (Devir et al., 1996; Morita et al., 2017). One suggested behavioral indicator of boredom is the motivation for general stimulation (Meagher, 2019) and sensation-seeking behaviors (Burn, 2017). We hypothesized that “refusals” may be a behavior associated with boredom, as they appear to be a sensation-seeking activity.

Self-grooming has been considered a comfort behavior that may have rewarding properties (Wilson et al., 1999; Boissy et al., 2007). As such, it has been cautiously discussed as a potential indicator of positive affective states (Napolitano et al., 2009; Mattiello et al., 2019); however, the literature appears contradictory. Decreased levels of self-grooming have been observed in sick compared with healthy cattle (Borderas et al., 2008; Fogsgaard et al., 2012) but also the opposite (Almeida et al., 2008). Increased self-grooming has also been reported in cows in more barren environments (Krohn, 1994; Di Grigoli et al., 2019) and in stressful conditions (Bolinger et al., 1997). The relationship between self-grooming and boredom is unknown; we hypothesized that it may be associated with other behavioral indicators of boredom.

The purpose of this study was to assess whether the provision of an additional behavioral opportunity, a novel object enrichment, would reduce behaviors hypothesized to be associated with boredom in cubicle-housed dairy cows. We also assessed how much time cows spent interacting with the novel object and the impact of this housing modification on the expression of self-grooming behavior. We hypothesized that the provision of an additional environmental enrichment would reduce idling behavior and milking refusals and increase the occurrence of self-grooming.

Ethical approval for the study was granted by The University of Nottingham, School of Veterinary Medicine and Science Ethical Review Committee, approval number 2697–190221. All methods were performed in accordance with the relevant guidelines and regulations.

Holstein cows were enrolled in the study (n = 71) and randomly assigned to 1 of 2 replicate study groups. Group 1 (mean ± SD): 35 Holstein cows with an average milk production of 44.93 ± 3.90 L of milk/d, averaging 163.5 ± 60.79 DIM, of parity 2.54 ± 1.44. Group 2: 36 Holstein cows with an average milk production of 39.30 ± 2.86 L of milk/d, averaging 141.9 ± 42.36 DIM, of parity 2.25 ± 1.32. The study was conducted at the Centre for Dairy Science Innovation, University of Nottingham, United Kingdom, which houses a 350-cow research dairy herd producing milk commercially. Study groups were consecutively housed in one 774.9 m^2^ sand-bedded cubicle building. Cows were milked robotically via a Lely A4 automatic milking system where they received additional concentrate feed, were fed a TMR daily at 0900 h, and had ad libitum access to fresh water and one automatic brush. Cows were managed according to the commercial management procedures at the Centre for Dairy Science Innovation.

The trial ran from September 28, 2020, to September 8, 2020 (group 1) and March 1, 2021, to April 4, 2021 (group 2). The 6-wk study period comprised an initial baseline week in which cows were housed in standard conditions (baseline wk 1). This was followed by a 3-wk treatment period, where continuous access to a novel object in the home pen was provided (intervention wk 1 to 3). Following the intervention weeks, the novel object was removed and cows spent 1 wk in standard housing conditions. Following this, a final baseline week was recorded (baseline wk 2). The novel object provided was an inflatable sailing buoy that was suspended by rope at cow shoulder height, in a loafing area at one end of the building. This object was arbitrarily chosen based on it being safe, indestructible, and interactive. It was not hypothesized to provide any specific behavioral outlet, but to provide diversity to the pen and an additional behavioral opportunity. A circle of a 2-m radius from the novel object was marked on the floor using paint. No other facilities such as lying or feeding areas were provided at the far end of the building where the novel object was situated to prevent cows from using this area for other purposes. Forty cubicles were available as lying areas for the 37 trial cows throughout both study periods.

Behavior was recorded using 4 Axis M10 network cameras (Axis Communications, Lund, Sweden). Cows were identified via a unique identification number that was applied using a water-based cattle tail paint twice a week. Video footage was analyzed by a single observer using Noldus Observer XT version 15 software. All statistical analyses were performed using RStudio version 4.0.3 using packages tidyverse (Wickham et al., 2019) and lme4 (Bates et al., 2015). Details of each element of analysis are provided later in this article, and inference was conducted through assessment of model parameter confidence intervals with a general significance threshold set at *P* < 0.05. All statistical models were assessed graphically to check for normality and homogeneity of residuals.

Idling was defined as a cow stood stationary, and may be looking around or changing position but with no other overt activity (De Rosa et al., 2009; Webb et al., 2017). Idling was evaluated using a scan sampling procedure with a 60-min scan interval. Sampling was carried out on Mondays and Fridays during baseline wk 1, intervention wk 1 to 3, and baseline wk 2, between 1100 and 0700 h, to avoid routine management procedures, meaning a total of 42 scans each week. For each scan, every cow was scored as idling or not idling. Results were accumulated to provide a sum for the number of idling events exhibited by all cows each day, and this was divided by the numbers of cows present. Final inference was made from a linear model with the outcome variable as the number of recorded behavioral events per cow per day.

Data were recorded continuously from the Lely robotic milking system for the entirety of both experimental replicates. Records included animal number, date, time of each visit, milk yield, and number of milking refusals. An unsuccessful milking attempt (refusal) was defined as when a cow entered the robot but was immediately released; this was a preset function of the robotic milking system and would occur when a cow entered the robot before a minimum time had elapsed since a previous milking. Depending on the individual cow yield and DIM, the minimum time allowed between milkings was 4.8 to 8 h. Data were recoded as a total number of refusals per cow per day and final inference made from a mixed effect linear model with number of refusals per cow per day as the outcome variable and a random term for cow to account for repeated measurements of refusals over time within cow (Bates et al., 2015). Since initial exploratory models revealed that model residuals displayed overdispersion (nonnormality), a transformed outcome variable was used [log_10_ (number of refusals per day + 1)] to ensure models met the required underlying assumptions.

Interactions with the novel object (sailing buoy) were evaluated using a single 24 continuous hours of footage per week during intervention wk 1 to 3, a total of 72 continuous h of footage analysis per study group. The 24-h period of continuous footage was selected such that no routine or unexpected farm interventions occurred (e.g., routine foot trimming or veterinary examinations), therefore representing a “normal” day for the cows. For group 1 this was Thursdays (October 8, 15, and 22 of 2020) starting from 0000 h and for study group 2 it was Tuesday, March 9, 2021, starting from 0900 h, Thursday, March 18, 2021, from 0000 h, and Tuesday, March 23, 2021, starting from 0900 h. The days evaluated for study group 2 differed from study group 1 to avoid interference from routine herd hoof trimming visits.

An interaction with the novel object was defined as physical contact with the novel object which started when any part of a cow's body came into contact with the buoy and ended when physical contact stopped for longer than 5 s. If the cow then contacted the object again following a 5 s break, this was defined as a new interaction. For every interaction, cow ID and length of interaction were recorded.

Self-grooming was defined as any licking, chewing, or scratching carried out by the cow either by mouth or by hoof directed at the cow's own body. To evaluate the occurrence of self-grooming events that were specifically linked to an interaction with the novel object, all self-grooming events that occurred within a 2 m radius of the novel object following an interaction with the object were identified and recorded during the same 24 continuous hours of footage sampled to measure object interaction, during intervention wk 1 to 3. To provide a comparison with the occurrence of self-grooming in baseline wk 1, all instances of self-grooming that occurred within the 2-m novel object area (but with no novel object present) during baseline wk 1 were recorded during the 24 h period of continuous footage (group 1: October 1, 2020; group 2: March 2, 2021). The probability that a cow would self-groom given that she was in the 2-m zone was calculated and compared between weeks. Cows were also categorized as either having self-groomed (on one or more occasion) or not, a binary variable, during each 1-h period of the 24 continuous hours of footage: this was coded as 1 (self-groomed) or 0 (did not groom) for all cows that were eligible having entered the 2-m zone. Final inference on the probability of self-grooming was made from a conventional mixed effect logistic regression model (Bates et al., 2015) that incorporated a random effect for cow to account for the repeated measurements of self-grooming over time within cow and therefore ensured a robust estimate of the conditional probability of self-grooming.

There was a significant reduction in the number of idling events of 0.19 events per cow per day during intervention wk 1 to 3 compared with the baseline wk 1 (*P* = 0.049). Following removal of the buoy, there was a significant increase in the number of idling events of 0.25 events per cow per day during baseline wk 2 compared with intervention wk 1 to 3 (*P* = 0.009). The mean number of idling events per cow per day is illustrated in Figure 1. Results of the final linear model are presented in Table 1.

**Figure 1:**
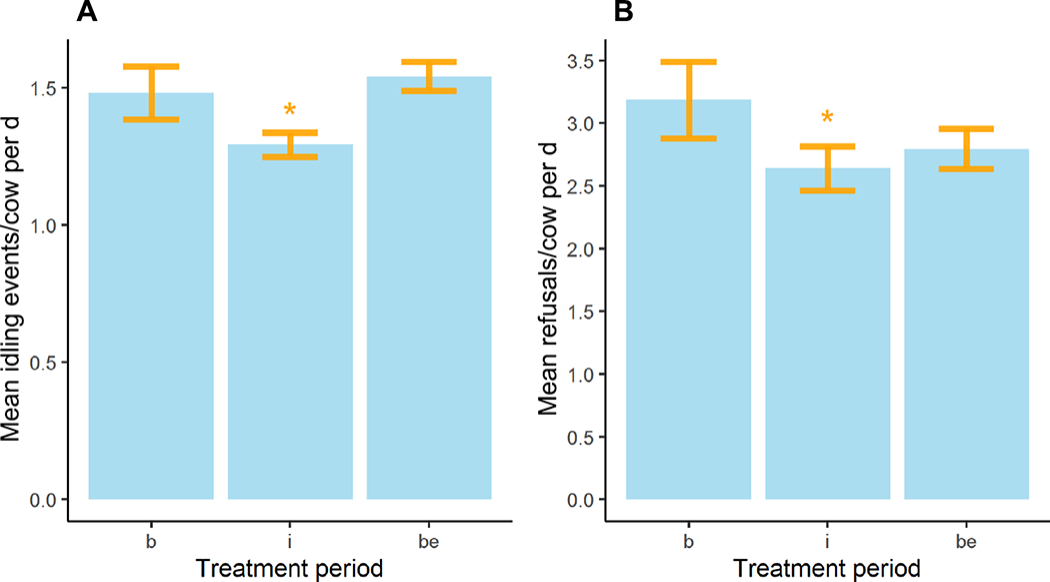
(A) Mean number of idling events per cow per day between trial periods. (B) Mean number of refusals per cow per day between trial periods. Significant differences between baseline wk 1 (b), baseline wk 2 (be), and intervention wk 1 to 3 are indicated (i): *P < 0.05. Standard errors are indicated in yellow.

**Table 1:** Results of the linear models used to assess boredom-associated behaviors during treatment periods

Model term	Coefficient	95% CI	*P*-value
Model 1: log_10_ (refusals per day + 1)^1^			
Treatment period			
Intercept	0.41		
Baseline wk 1	Referent		
Intervention wk 1–3	−0.06	−0.09 to −0.03	<0.001
Baseline wk 2	−0.03	−0.06 to 0.01	0.142
Model 2: idling (events per cow per day)^2^			
Treatment period			
Intercept	1.48		
Baseline wk 1	Referent		
Intervention wk 1–3	−0.19	−0.38 to −0.001	0.049
Baseline wk 2	0.06	−0.17 to 0.29	0.588
Model 3: self-grooming (yes or no)^3^			
Treatment period			
Intercept			
Baseline wk 1	Referent		
Intervention wk 1–3	Odds ratio = 4.19	2.76 to 6.36	<0.001

1 The outcome variable for model 1 was the log_10_ of the total refusals + 1 per cow per day.

2 The outcome variable for model 2 was the average number of idling events per cow per day.

3 The outcome variable for model 3 was whether a cow self-groomed (yes or no) within the 2-m enrichment area.

The number of refusals per cow per day followed an overdispersed, right-skewed distribution with a small number of cows having a relatively high numbers of refusals. A log (base 10) transformation was used to normalize the data and allow robust comparison between groups. The distributions of the mean number of refusals per cow per day are illustrated in Figure 1. Results from the mixed effect linear model with log_10_(refusals per day + 1) as the outcome are provided in Table 1. There was a significant reduction in daily cow refusals (equating to a reduction of 0.5 refusals per day) during the weeks when the novel object was present compared with the baseline wk 1 (*P* < 0.001) and baseline wk 2 (*P* = 0.02).

During the time of sampled video footage, in baseline wk 1, there were 142 separate entries to the 2-m enrichment zone and self-grooming occurred at least once during 42 of these visits (29.58%). During intervention wk 1 to 3, respectively, the number (%) of entries where self-grooming occurred was 150/236 (63.56%), 97/161 (60.25%), and 79/123 (64.22%). The proportion of individual cows that self-groomed within the 2-m enrichment zone during baseline wk 1 was 0.49 ± 0.06. The proportion of individual cows that self-groomed during intervention weeks was 0.84 ± 0.05, 0.77 ± 0.05, and 0.83 ± 0.05 for wk 1, 2, and 3, respectively. Results of the mixed effects models showed that having accounted for repeated measurements of grooming within cow, the odds of a cow self-grooming were significantly increased during intervention wk 1 to 3 compared with baseline wk 1 (odds ratio = 4.19, 95% CI 2.76–6.36, *P* < 0.001). Based on this model the calculated adjusted probability of self-grooming in baseline wk 1 was 0.29 and during the intervention period was 0.64, 0.61, and 0.65 during wk 1, 2, and 3, respectively.

During intervention wk 1 to 3, one or more cows interacted with the buoy at least once during 66.5 of the 72 h of continuous video footage. During these weeks, cows spent a mean of 12.09 ± 1.21 min per day interacting with the buoy during wk 1, 7.18 ± 0.96 min during wk 2, and 4.64 ± 0.64 min during wk 3. Cows spent significantly less time using the buoy during intervention wk 2 (*P* < 0.01, 95% CI −7.62 to −2.19) and 3 (*P* < 0.01, 95% CI −10.21 to −4.69) compared with intervention wk 1; however, the proportion of cows that continued to interact with the buoy throughout the study remained high (intervention wk 1: 0.92 ± 0.03, wk 2: 0.89 ± 0.04, and wk 3: 0.83 ± 0.04).

Almost all cows from 2 separate replicates repeatedly interacted with the novel object throughout the intervention period. It seems reasonable to interpret this interaction as a positive experience by the cows, given that cows will actively avoid situations they associate with negative events (Munksgaard et al., 1997; Pajor et al., 2000). In addition, the time that cows spent using the novel object was similar to how much time cows spend using brushes (DeVries et al., 2007; Mandel et al., 2017), which are widely regarded to be a positive behavioral opportunity for cows (Mandel et al., 2016; McConnachie et al., 2018). Although the use of enrichment declined across study weeks, which suggests a need to understand how cows would use additional enrichment over time, this habituation appears to be a consistent response to the provision of novel stimuli in general (Trickett et al., 2009; Van Os et al., 2021). The behavioral changes observed in response to provision of a simple novel object in the present study suggest that other enrichment opportunities which are used more, for example, outdoor space (Russell et al., 2023), could have a greater impact on behavior.

Fewer cows exhibited idling behavior when the novel object was present compared with when it was not, which is consistent with other studies that have shown decreased levels of wakeful inactivity in more stimulus-diverse environments in cattle (Webb et al., 2017; Hintze et al., 2020). The use of wakeful inactivity as a potential marker of negative affective states is supported by its correlation with other characterizable symptoms of depression (Fureix et al., 2016; MacLellan et al., 2022) and observed reduction through administration of antidepressants (Kudryavtseva et al., 1991; Fureix et al., 2022). Similarly, it has been correlated with heightened interest in both rewarding and aversive stimuli consistent with boredom (Meagher and Mason, 2012). Reductions in idling observed could simply be a reflection of cows being more engaged in other behavioral activities without changes in affective state. Further studies would be required to elucidate this; however, our results suggest that idling may be a behavioral indicator of boredom in cows.

This is the first study to suggest that automatic milking refusals may be a potential behavioral indicator of boredom in dairy cows and reduced refusals occurred when cows were provided with additional environmental enrichment. The anticipation of reward and having control over a positive outcome are 2 cognitive processes that may be associated with positive emotions in animals (Boissy and Lee, 2014). Use of the robot may provide opportunity for these experiences, particularly when other behavioral opportunities are limited.

In contrast to the decline in idling and refusals, results demonstrated increased self-grooming behavior associated with use of enrichment. Cows could choose to interact with or avoid the novel object, and most cows repeatedly used it, suggesting it unlikely to represent a stressful situation. Increased self-grooming has previously been reported in dairy cows in response to novelty (Herskin et al., 2004). Self-grooming is also linked to hormones released following stress or arousal (Niesink and Van Ree, 1989; Spruijt et al., 1992); therefore, it may be plausible for the behavior to be a response to changes in stress or arousal, which could be valenced in either direction. Self-grooming appears to be sensitive to environmental and physiological conditions; validated indicators of positive states and stress should be used alongside it to allow better interpretation of changes in this behavior.

In conclusion, this intervention study addressed an important research gap and observed notable changes to cow behavior, which suggests that idling and refusals may provide potential behavioral indicators of boredom in cows. The provision of additional forms of enrichment may be beneficial to housed dairy cows and would be a beneficial line of further research.
